# Supplementary material for: The Effects of Long Duration Spaceflight on Sensorimotor Control and Cognition
Source: Front Neural Circuits. 2021 Oct 26;15:723504. doi: 10.3389/fncir.2021.723504 (PMC8577506; doi:10.3389/fncir.2021.723504)
Supplement: Supplementary file 1 [file Data_Sheet_1.docx]

**The effects of long duration spaceflight on sensorimotor control and cognition**

**Supplementary Material**

**GD Tays^1^, KE Hupfeld^1^, HR McGregor^1^, AP Salazar^1^, YE De Dios^2^, NE Beltran^2^, PA Reuter-Lorenz^3^, IG Kofman^2^, SJ Wood^4^, JJ Bloomberg^4^, AP Mulavara^2^,** ***RD Seidler^1,5^**

^1^ Department of Applied Physiology and Kinesiology, University of Florida, Gainesville, FL, USA

^2^ KBR, Houston, TX, USA

^3^ Department of Psychology, University of Michigan, Ann Arbor, MI

^4^ NASA Johnson Space Center, Houston, TX, USA

^5^ Norman Fixel Institute for Neurological Diseases, University of Florida, Gainesville, FL

***Correspondence:**

Rachael Seidler, PhD

rachaelseidler@ufl.edu

(352) 294-1722

Figure S1


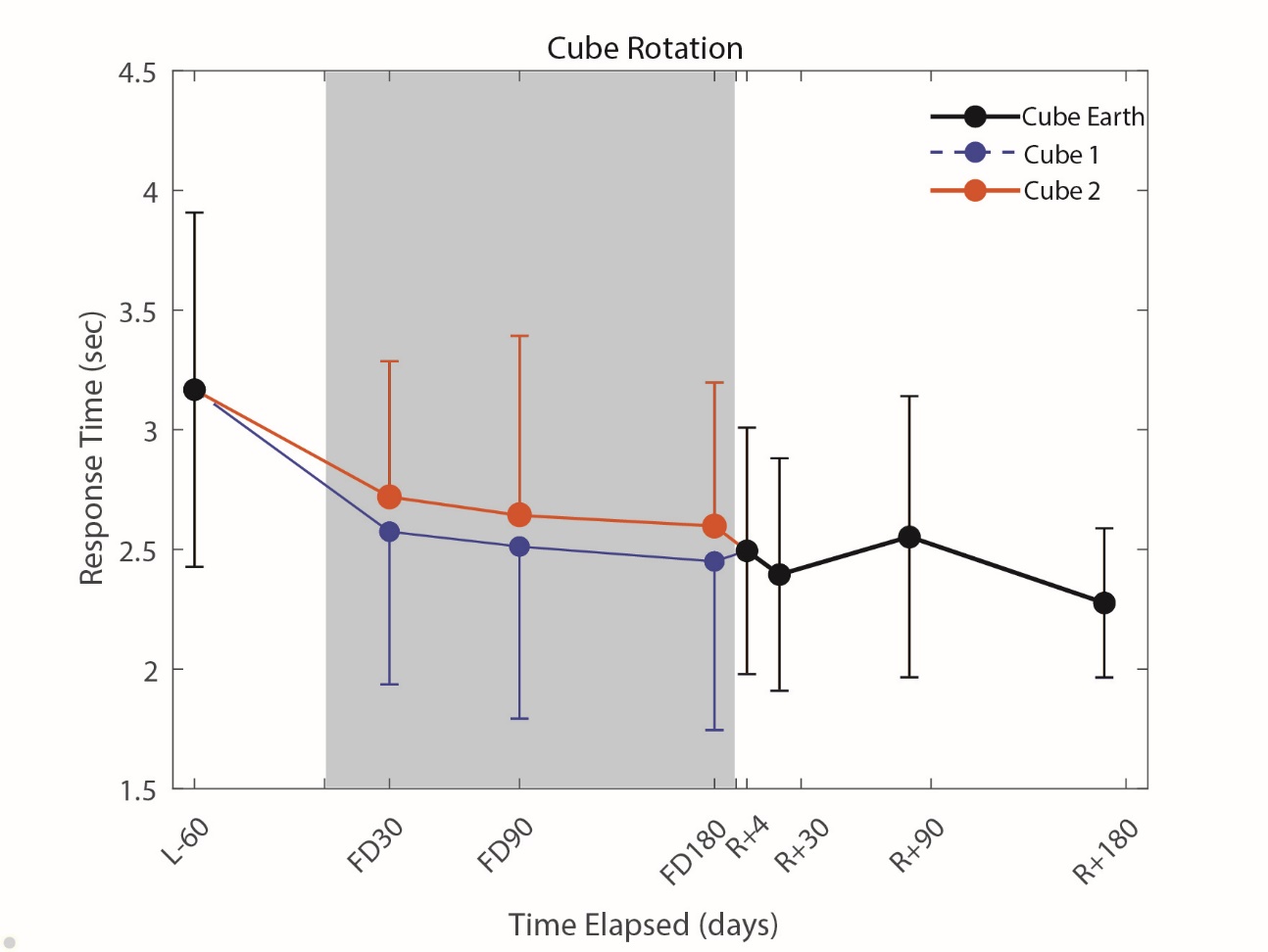


Figure S1. Cube rotation performance changes from pre- to in-flight post-flight. The black line depicts performance on Earth, the blue line illustrates performance while in microgravity free floating, and the orange depicts performance while in microgravity with the feet looped into the “floor” and in a “seated” position. Response time improved (p=0.004) from pre- to post-flight. There was a trend level difference of a faster response time on cube 2 as compared to cube 1 in-flight of 141 ms on average(p=0.093).
